# Comprehensive Computational Analysis of Protein Phenotype Changes Due to Plausible Deleterious Variants of Human SPTLC1 Gene

**DOI:** 10.22088/IJMCM.BUMS.8.1.67

**Published:** 2019-04-23

**Authors:** Tayyaba Sadaf, Peter John, Attya Bhatti

**Affiliations:** *Department of Health Care Biotechnology, Atta-ur-Rahman School of Applied Biosciences (ASAB), National University of Sciences and Technology (NUST), Islamabad, Pakistan.*

**Keywords:** Single nucleotide polymorphisms, computational, deleterious, variants, bioinformatics tools

## Abstract

Genetic variations found in the coding and non-coding regions of a geneare known to influence the structure as well as the function of proteins. Serine palmitoyltransferase long chain subunit 1 a member of α-oxoamine synthase family is encoded by *SPTLC1* gene which is a subunit of enzyme serine palmitoyltransferase (SPT). Mutations in *SPTLC1* have been associated with hereditary sensory and autonomic neuropathy type I (HSAN-I). The exact mechanism through which these mutations elicit protein phenotype changes in terms of structure, stability and interaction with other molecules is unknown. Thus, we aimed to perform a comprehensive computational analysis of single nucleotide polymorphisms (SNPs) of *SPTLC1* to prioritize a list of potential deleterious SNPs and to investigate the protein phenotype change due to functional polymorphisms. In this study, a diverse set of *SPTLC1* SNPs were collected and scrutinized to categorize the potential deleterious variants. Our study concordantly identified 21 non- synonymous SNPs as pathogenic and deleterious that might induce alterations in protein structure, flexibility and stability. Moreover, evaluation of frameshift, 3’ and 5’ UTR variants shows c.*1302T> Gas effective. This comprehensive *in silico* analysis of systematically characterized list of potential deleterious variants could open avenues as primary filter to substantiate plausible pathogenic structural and functional impact of variants.

Sphingolipids belong to a diverse family of cellular lipids that perform fundamental functions both as membrane components and as signaling molecules (1). Cells obtain sphingolipids intrinsically by *de novo* biosynthesis and extrinsically by up- take and reusing the exogenous sphingolipids (1). An endoplasmic reticulum-confined enzyme, serine palmitoyltransferase (SPT), is a pyridoxal 5'- phosphate dependent multimeric enzyme, which acts as a vital player for *de novo* biosynthesis of sphingolipids. This enzyme catalyzes the foremost step of sphingolipid metabolism i.e., the condensation of L-serine and palmitoyl coenzyme (CoA) for producing 3-ketodihydrosphingosine (KSD) (2, 3). The activity of SPT in *de novo* sphingolipid biosynthesis pathway is required for various normal cellular functions including the survival of adipocyte cells. The decreased *de novo* sphingolipid biosynthesis inside adipocytes leads to adipocyte death, adipose tissue remodeling, and metabolic disorder (4).

An important SPT subunit, SPT long chain subunit 1 encoded by *SPTLC1* gene is the member of α-oxoamine synthase family (5). It is mapped to chromosome 9q22.1-q22.3, and contains 15 exons that encode for a protein with 473 amino acid residues (6). The structure and function of SPT is usually disturbed by mutations in *SPTLC1* gene, which occur at amino acids that are highly conserved throughout various species (7). Mutations in *SPTLC1* have been associated with hereditary sensory and autonomic neuropathy type I (HSAN-I) (6, 8). HSAN-I is an autosomal predominant dynamic degenerative hereditary disorder of peripheral sensory neurons characterized by dorsal root ganglia (DRG) and motor neurons degeneration. It is the most common subtype of HSAN or hereditary sensory neuropathy (HSN). In HSAN-I, the enzymatic selectivity of mutant SPT is lost and L-alanine is utilized as an alternative substrate, which results in the formation of atypical and neurotoxic 1-deoxy-spingolipids (9, 10). This promiscuous enzymatic activity of mutant SPT is suggested to be the pathological reason of HSAN-I (11, 12). A noticeable rise in endoplasmic reticulum (ER) stress has also been observed in HSAN-I patient cells, expressing the p.V144D mutant SPTLC1 protein as compared to cells of healthy controls (13). The protein modifications reflect the altering cellular events that bring about HSAN-I. Recently, a notable change in the expression of a group of proteins in the mitochondria and ER has been detected in SPTLC1 p.V144D mutant lymphoblasts (14-16). Notably, identified changes also exhibited in the p.C133W and p.C133Y mutations (17).

During recent years, there has been extensive consideration in associating the genetic variations to protein phenotype changes. However, determining the disease-associated missense mutations had been a challenging task for genetic disorder research. Owing to the significance of *SPTLC1* mutations and its subsequent link with a spectrum of clinical pathologies, this study has intended to investigate the disease causal mutations in exonic and regulatory regions (5’ and 3’ UTRs) to develop the predictions and facilitate their pathogenic characterization based on their impact to structure and function of SPTLC1 protein. Thus, we implemented computational approach for screening the possible detrimental mutations of *SPTLC1* and computationally analyzed structural and functional impact of screened potential mutations.

## Material and methods

**Collection of dataset**

**Fig. 1:**
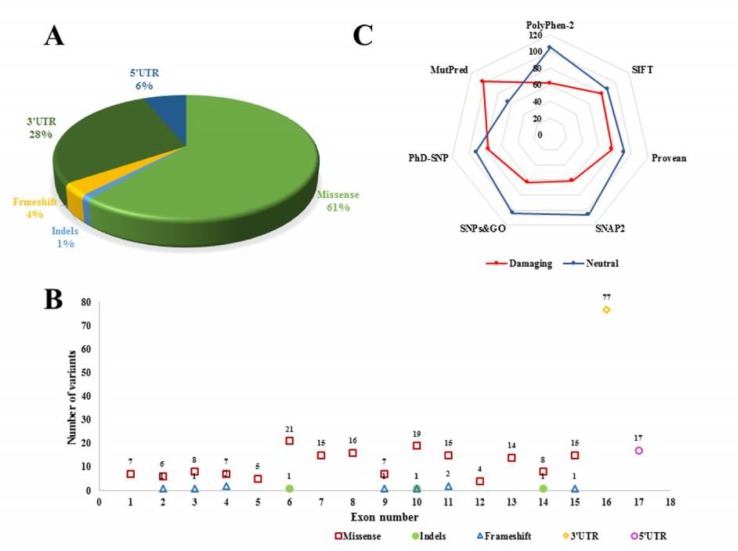
Distribution of SPTLC1 nucleotide variants. A: pie chart representing different classes of mutations; B: scatter plot representing the number of missense, indels, frameshift mutations per exon and number of 3’UTR and 5’UTR variants; C: radar chart representing the total number of pathogenic and neutral SPTLC1 variants by each prediction program

The *SPTLC1* polymorphisms data belong to NM_006415.2 transcript and NP_006406.1 amino acid sequence was mined from databases including NCBI (National Centre for Biotechnology Information) affiliated dbSNP(18) and exome variant server (Server EV. NHLBI GO exome sequencing project (ESP)). Concerned protein sequence and information was retrieved from Ensembl (19) (ENSG00000090054; ENSP00000 262554), OMIM (Online Mendelian Inheritance in Man) (20) and UniProt (UniProt Consortium, 2015) (O15269), that provide ample high-quality sequence and functional information of protein for our computational analysis. Redundant mutations obtained from various sources were eliminated to reform the data. Based on variants nature and position, data was classified as missense, insertion and deletions, frameshift and untranslated regions (Fig. 1A).

**Analysis of variants at genomic level**

**Prediction of nsSNPs having structural and functional impact**

To predict important SNPs influencing a protein upon substitution functionally, servers like Sorting Intolerant from Tolerant (SIFT), Polymorphism Phenotyping v2 (PolyPhen-2), Protein Variation Effect Analyzer (PROVEAN) and MutPred were used. These servers provide rapid analysis of variants supporting high-throughput investigation at genetic and protein level. Firstly, the variants were assessed by a sequence homology-based program SIFT (21-23). If the score of the variant was less than a chosen threshold (≤0.05), the variant was classified as deleterious and vice versa. Physiochemical differences, evolutionary conversation, and substitution proximity to the structural level alterations of protein upon substitution were identified by PolyPhen-2 (24). The variant was categorized as “probably damaging” by PolyPhen-2, if the position-specific independent count (PSIC) score was 0.99-1.00, and “possible damaging” if the score was 0.50-0.99, and the rest were categorized as “benign” (with no phenotypic influence). Biological functional changes of a protein due to a variant were also computed by PROVEAN that worked on sequence clustering and alignment-based scoring. The variant was classified as deleterious if the prediction score was <-2.5 (25, 26), according to PROVEAN program. To examine whether the molecular variance was involved in insurgence of human diseases, the impact of variants was also estimated by web-based tool MutPred (27).

**Indels, frameshift and UTR variants analysis**

The detrimental nature of insertions, deletions and frameshift mutations were predicted by SIFT Indel Classifier that requires comma separated list of chromosome coordinates, orientation (1, -1) and indels as input (28). Functionally important indels were also filtered by PROVEAN. The indels were considered deleterious if the score was <=-2.5 and neutral if the variant score was > -2.5 (25, 26). Functional sequence pattern positioned in 5’ and 3’ UTR sequences were collected from dbSNP (18) and specialized untranslated regions of eukaryotic mRNAs databases: UTRdb and UTR site (29, 30). These variants were analyzed by UTR specific tool UTRScan. User submitted sequences were carefully searched by UTRScan for any functional elements or patterns endorsed by UTRsite and UTR database.

**Analysis of variants at structural level**

**Modeling of SPTLC1 protein structure**

The human SPTLC1 protein sequence comprising 473 amino acid residue was subjected to SWISS-MODEL (31-34) for homology modeling. Evaluation of modeled structure was carried out using ERRAT (35), RAMPAGE (36) and ProSA-web (37) servers. The structure was passed through energy minimization step to remove the internal constraints with GROMOS96 implementation of Swiss-PdbViewer 4.1.0 after adding hydrogen atoms (38).

**Analysis of protein characteristics properties**

MUpro server was used to find out the effect of non-synonymous SNPs (nsSNPs) on protein stability. The predicted score less than 0 shows decrease in protein stability due to the mutation; contrariwise, a score greater than 0 refers to an increase in protein stability (39). Solvent acce-ssibility of structures was predicted by an artificial neural network-based program NetSurfP-1.1 (40) and Predict Protein (41). For approximating residue specific quality of protein structure prediction and the inherent B-factor profile of all residues along the chain by combining local structure assembly variations with sequence- and structure-based profilingResQ server was used (42).

**Functional analysis of mutations**

Multi-scale binding pockets on SPTLC1 protein surface were explored by GHECOM 1.0: Grid-based HECOMi finder server (43). Functional association of SPTLC1 protein was critically assessed using the Search Tool for the Retrieval of Interacting Genes (STRING) v10 database (44). Protein-Protein interaction of SPTLC1 including both physical and functional associations based on known interactions (curated and experimentally determined), predicted interactions (gene neighborhood, gene fusions and gene co-occurrence), text mining, co-expression and protein homology was identified. The edges of network represent the association between nodes (interacting proteins).

**Protein-protein docking simulation**

A flexible protein docking approach, the HADDOCK (High Ambiguity Driven protein-protein DOCKing) version 2.2 (45) was used to perform modeling of biomolecular complex: SPTLC1 with its highest interacting partner. The identification of active and passive residues of interacting biomolecules was performed by CPORT (46).

## Results

**Mutation spectrum of** ***SPTLC1*** **gene**

The examined gene comprises a total of 273 human SNPs belonging to different classes of mutations including synonymous and non-synonymous. Among all the included mutations in our study, missense mutations seemed to be the most abundant mutations with n =168 (61.5%) when compared to indels (n = 3), frameshift (n = 9), and UTRs (n = 94; 34.4%) (Fig. 1A). Noticeable uneven distribution of mutations in exons is represented in Fig. 1B.

**Table 1:** List of selected SPTLC1 missense variants with their corresponding exon, chromosome position and protein variants with predicted scores by SIFT, PolyPhen-2, PROVEAN, MutPred and MUpro

**S. No.**	**NT** **Variant**	**Exon**	**Chr. position**	**PRO Variant**	**PolyPhen-2**		**SIFT**		**PROVEAN**		**Mut-Pred**	**MUpro**
					**Pred**	**Score**	**Pred**	**Score**	**Pred**	**Score**	**Pred PR**	
**1**	310G>A	4	92080914	104A>T	pr dmg	0.998	dmg	0.01	del	-3.079	0.779	-1.2661182
**2**	325C>G	4	92080899	109L>V	pr dmg	0.989	dmg	0.01	del	-2.901	0.856	-1.0533436
**3**	398G>A	5	92080045	133C>Y	pr dmg	1	dmg	0	del	-10.725	0.839	-0.282728
**4**	399T>G	5	92080044	133C>W	pr dmg	1	dmg	0	del	-10.726	0.853	-0.31455734
**5**	431T>A	6	92068095	144V>D	pr dmg	0.998	dmg	0	del	-6.207	0.871	-1.9523302
**6**	457G>A	6	92068069	153A>T	pos dmg	0.882	dmg	0	del	-3.584	0.901	-1.3441989
**7**	481G>A	6	92068045	161A>T	poss dmg	0.87	dmg	0	del	-3.411	0.796	-1.2131073
**8**	485T>G	6	92068041	162I>S	pr dmg	0.996	dmg	0	del	-5.365	0.772	-2.3519006
**9**	524T>C	6	92068002	175I>T	pr dmg	0.999	dmg	0	del	-4.698	0.784	-1.8899242
**10**	563A>C	7	92059306	188D>A	pr dmg	0.988	dmg	0.01	del	-7.484	0.895	-0.97234279
**11**	743A>G	8	92055442	248Y>C	benign	0.053	dmg	0	del	-7.668	0.771	-0.67134313
**12**	832T>G	9	92050016	278S>A	poss dmg	0.59	TOL	0.07	del	-2.641	0.845	-1.2437183
**13**	929C>G	10	92047668	310A>G	benign	0.006	dmg	0.02	del	-2.824	0.832	-1.4961316
**14**	946G>A	10	92047651	316G>S	pr dmg	0.993	dmg	0.01	del	-5.191	0.927	-1.3775049
**15**	952T>A	10	92047645	318C>S	pr dmg	0.989	TOL	0.05	del	-8.278	0.808	-0.56363416
**16**	992C>T	11	92047261	331S>F	benign	0.222	dmg	0.03	del	-4.533	0.759	-0.67561754
**17**	992C>A	11	92047261	331S>Y	poss dmg	0.454	dmg	0	del	-4.50	0.825	-0.97224916
**18**	1055C>T	11	92047198	352A>V	benign	0.066	dmg	0.01	del	-2.909	0.857	-0.63107997
**19**	1160G>C	13	92038342	387G>A	benign	0.41	dmg	0.03	del	-3.117	0.817	0.07064886
**20**	1334G>A	15	92032553	445R>Q	pr dmg	0.998	dmg	0.01	del	-3.245	0.88	-1.3868538
**21**	1333C>T	15	92032554	445R>W	pr dmg	1	dmg	0	del	-6.841	0.874	-1.2548201

NT: nucleotide; Chr: chromosome; PRO: protein; Pred: prediction; Accu: accuracy; PR: probability; poss: possibility; dmg: damaging; TOL: tolerant; N: neutral; DIS: disease.

**Analysis at genomic level**

**Analysis of deleterious missense mutations**

Among the 168 missense mutations, SIFTanalysis revealed 80 (47.6%) nsSNPs as “damaging” or “intolerant” having a tolerance index score of ≤0.05, while 88 (52.3%) mutations were “tolerant” with > 0.05 score (Fig. 1C). Out of 80 damaging mutations, 33 (41.25%) and 24 (30%) nsSNPs were “extremely-intolerant” with 0.00 and 0.01 score, respectively and 23 (28.75%) nsSNPs were just “intolerant”. According to PolyPhen-v2 prediction, a total of 63 (37.5%) nsSNPs were expected to be damaging. Of which, 36 nsSNPs were “probably damaging” with score ranging from 0.99 to 1.00, and 27 were “possibly damaging” with score ranging from 0.5 to 0.9, and the remaining 105 nsSNPs were classified as benign. A total of 77 (45.8%) mutations were predicted deleterious and 91 (54.1%) were neutral by PROVEAN. Among all the deleterious mutations 54 (70.1%) were least deleterious, 23 (29.8%) were deleterious with score < -5.0, of which 2 mutations (p.C133CY and p.C133W) were deleterious with score < -10.0. About 104 (61.9%) and only 28 (16.66%) nsSNPs with > 0.5 and 0.75 probability score were predicted as disease associated mutations by MutPred. However, the concordant analysis predicted 21 mutations mentioned in Table 1 as potential predicted mutations that can be deleterious. Protein stability analysis by MUpro revealed that all the selected mutants would decrease the stability except p.G387A as the predicted score of all other mutants was less than zero (Table 1).

**Table 2:** SIFT indel classifier and PROVEAN prediction analysis for indels and frameshift variants

**Nucleotide variant**	**Coordinates**	**Subs. type**	**Exon**	**AA variant**	**Clin. sig.**	**PROVEAN**		**SIFT**	
						**Score**	**Pred.**	**Score**	**Pred.**
c.139delC	92112481	FS-del	2	Q47Kfs	NA	-	-	0.858	dam
c.174delA	92108826	FS-del	3	E59Nfs	NA	-	-	0.858	dam
c.281_282delTG	92080942:92080943	FS-del	4	V94Gfs	NA	-	-	0.858	dam
c.277_278insA	92080946:92080947	FS-ins	4	T93Nfs	NA	-	-	0.858	dam
c.452_454delGCC	92068072:92068074	del	6	R151del	NA	-12.837	dele	0.858	dam
c.804_805insTA	92050043:92050044	FS-in	9	A269Terfs	NA	-	-	0.858	dam
c.895_897delGAT	92047700:92047702	del	10	D299del	NA	-8.167	dele	0.529	dam
c.963_964insG	92047633:92047634	FS-ins	10	S322Vfs	NA	-	-	0.858	dam
c.1031delT	92047222	FS-del	11	L344Rfs	NA	-	-	0.858	dam
c.1029_1030delCC	92047223:92047224	FS-del	11	L344Vfs	NA	-	-	0.858	dam
c.1305_1307delAGA	92034831:92034833	del	14	E436del	NA	-1.925	N	0.858	dam
c.1361_1362delAG	92032525:92032526	FS-del	15	E454Gfs	NA	-	-	0.783	Dam

Subs. Type: substitution type; FS: frameshift; del: deletion; ins: insertion; Clin.sig.:clinical significance; Pred.: prediction; dele: deleterious; N: neutral; dam: damaging

**Fig. 2:**
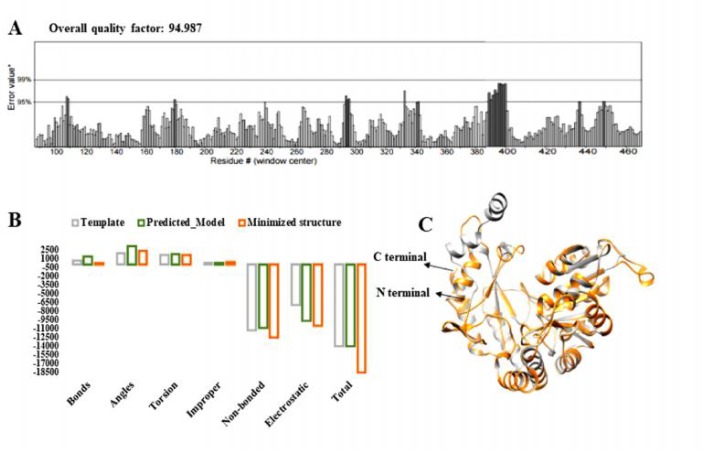
Selected human SPTLC1 predicted protein structure evaluation and energy value representation. A: ERRAT indicates 94.987 overall quality factor; B: colored bars used for representing the computed energy values in KJ/mol of template and predicted model (grey color for template used for structure prediction, green color for predicted model before energy minimization, and orange color for predicted model after energy minimization step); C: superimposition of template 3a2b (grey) and predicted SPTLC1 structure (orange red) shows high structural similarity.

**Table 3:** UTRScan prediction result for 3’UTR variants of SPTLC1 protein (Transcript ID:NM_006415.2).

**S.No.**	**rs ID**	**Position**	**Prediction**	**S.No.**	**rs ID**	**Position**	**Prediction**
1	rs758071979	c.*10C>T	-	40	rs115637483	c.*490A>G	-
2	rs200727312	c.*11G>A	-	41	rs531407417	c.*494T>C	-
3	rs778790410	c.*13G>A	-	42	rs74939390	c.*525G>T	uORF [519,608]
4	rs756960214	c.*20G>C	uORF [17,109]	43	rs144733313	c.*569G>A	uORF [519,608]
5	rs753599241	c.*23T>C	uORF [17,109]	44	rs367609260	c.*581T>C	uORF [519,608]
6	rs867197507	c.*28C>T	uORF [17,109]	45	rs537125477	c.*590T>G	-
7	rs374737655	c.*31C>T	uORF [17,109]	46	rs773137233	c.*614C>T	-
8	rs760602474	c.*38C>T	uORF [17,109]	47	rs576072015	c.*654A>G	-
9	rs370307230	c.*39G>A	uORF [17,109]	48	rs765100762	c.*657A>C	-
10	rs202080725	c.*46A>C	uORF [17,109]	49	rs761445360	c.*664C>G	-
11	rs550740752	c.*46G>A	uORF [17,109]	50	rs189417944	c.*670G>A	-
12	rs763262266	c.*50T>C	uORF [17,109]	51	rs866982133	c.*711T>G	uORF [705,782]
13	rs773269599	c.*58C>T	uORF [17,109]	52	rs142008725	c.*713A>C	uORF [705,782]
14	rs535778954	c.*60C>T	uORF [17,109]	53	rs879644362	c.*745C>G	uORF [705,782]
15	rs73653020	c.*61G>A	uORF [17,109]	54	rs768395365	c.*750C>T	uORF [705,782]
16	rs777118329	c.*68A>G	uORF [17,109]	55	rs568268325	c.*809T>C	-
17	rs1131864	c.*78C>T	uORF [17,109]	56	rs527344506	c.*822C>T	-
18	rs769349062	c.*95C>T	uORF [17,109]	57	rs374347262	c.*828T>G	uORF [827,1057]
19	rs1131866	c.*102A>G	uORF [17,109]	58	rs760223808	c.*864C>T	uORF [827,1057]
20	rs7024575	c.*112G>A	-	59	rs535318963	c.*867G>A	uORF [827,1057]
21	rs189582528	c.*124A>G	-	60	rs570805058	c.*875A>T	uORF [827,1057]
22	rs771433261	c.*133A>G	uORF [125,250]	61	rs570164486	c.*916A>G	uORF [827,1057]
23	rs745563960	c.*144A>G	uORF [125,250]	62	rs775237786	c.*932A>G	uORF [827,1057]
24	rs544879549	c.*147G>A	uORF [125,250]	63	rs771458551	c.*983T>C	uORF [827,1057]
25	rs184220566	c.*178T>A	uORF [125,250]	64	rs559735773	c.*1009G>T	uORF [827,1057]
26	rs552433019	c.*190A>C	uORF [125,250]	65	rs530944752	c.*1015G>A	uORF [827,1057]
27	rs753700526	c.*196A>G	uORF [125,250]	66	rs367968859	c.*1034T>C	uORF [827,1057]
28	rs377023278	c.*217T>A	uORF [125,250]	67	rs766363634	c.*1046T>C	uORF [827,1057]
29	rs531033514	c.*228A>G	uORF [125,250]	68	rs145019674	c.*1052A>G	uORF [827,1057]
30	rs563505829	c.*272A>G	-	69	rs77041650	c.*1067C>T	uORF [1063,1158]
31	rs766183581	c.*290T>C	ORF [281,376]	70	rs548652432	c.*1068A>G	uORF [1063,1158]
32	rs542032121	c.*320G>A	ORF [281,376]	71	rs142740904	c.*1154T>C	uORF [1063,1158]
33	rs564259149	c.*334C>G	ORF [281,376]	72	rs112076327	c.*1170T>C	-
34	rs529884120	c.*401C>A	-	73	rs760602744	c.*1209G>A	uORF [1177,1242]
35	rs372012368	c.*402A>T	-	74	rs541013337	c.*1221C>T	uORF [1177,1242]
36	rs7944	c.*445A>G	uORF [410,478]	75	rs562277733	c.*1226G>T	uORF [1177,1242]
37	rs868416931	c.*451G>T	uORF [410,478]	76	rs530126189	c.*1230G>A	uORF [1177,1242]
38	rs541284488	c.*483A>G	-	77	rs7035964	c.*1302T>G	CPE [1290,1339], IRES [1243,1339], uORF [1265,1333], PAS [1300,1339]
39	rs181586912	c.*488G>T	-				

**Fig. 3:**
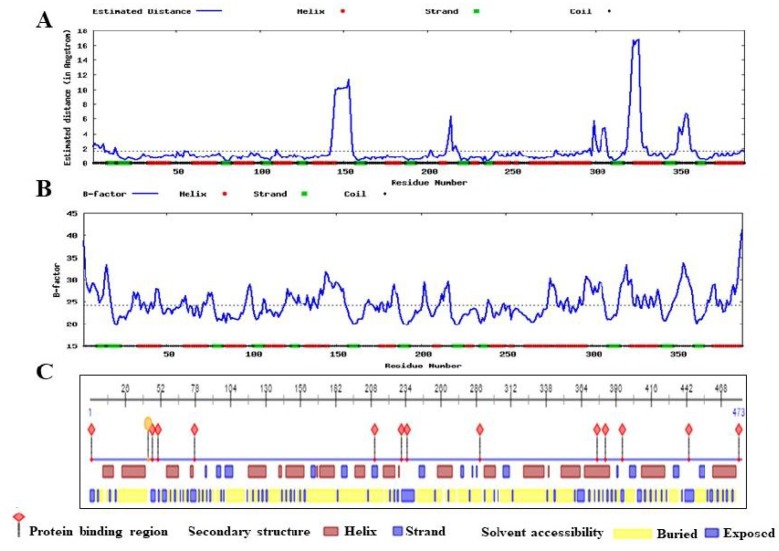
Analysis of protein properties. A: secondary structure and solvent accessibility analysis by PredictProtein; B: the local quality defined as the distance deviation (in Angstrom) between residue positions in the model and the native structure; C: stability of different parts of the structure in terms of beta factor by ResQ server

**Table 4:** UTRScan prediction result for 5’UTR variants of SPTLC1 protein.

**S.No.**	**rs ID**	**Transcript ID**	**Position**	**Prediction**
**1**	rs750255730	NM_006415.2	c.-3A>G	**-**
**2**	rs758217796	NM_006415.2	c.-6C>A	**-**
**3**	rs746676272	NM_006415.2	c.-7G>T	**-**
**4**	rs754378890	NM_006415.2	c.-10G>C	**-**
**5**	rs780821663	NM_006415.2	c.-19C>T	**-**
**6**	rs558203491	NM_006415.2	c.-27C>T	**-**
**7**	rs770382920	NM_006415.2	c.-28C>A	**-**
**8**	rs201897322	NM_006415.2	c.-29A>C	**-**
**9**	rs773682043	NM_006415.2	c.-34T>C	**-**
**10**	rs866449132	NM_006415.3	c.-39C>A	**-**
**11**	rs749631140	NM_006415.3	c.-49A>G	**-**
		NM_006415.3	c.-49A>T	**-**
**12**	rs774659397	NM_178324.2	c.-51G>A	**-**
**13**	rs55740103	NM_006415.3	c.-64T>C	**-**
**14**	rs552690353	NM_178324.2	c.-70C>T	**-**
**15**	rs184693119	NM_006415.3	c.-76T>C	**-**
**16**	rs111298150	NM_006415.3	c.-96C>T	**-**
**17**	rs557306141	NM_178324.2	c.-103G>T	**-**

**Fig. 4:**
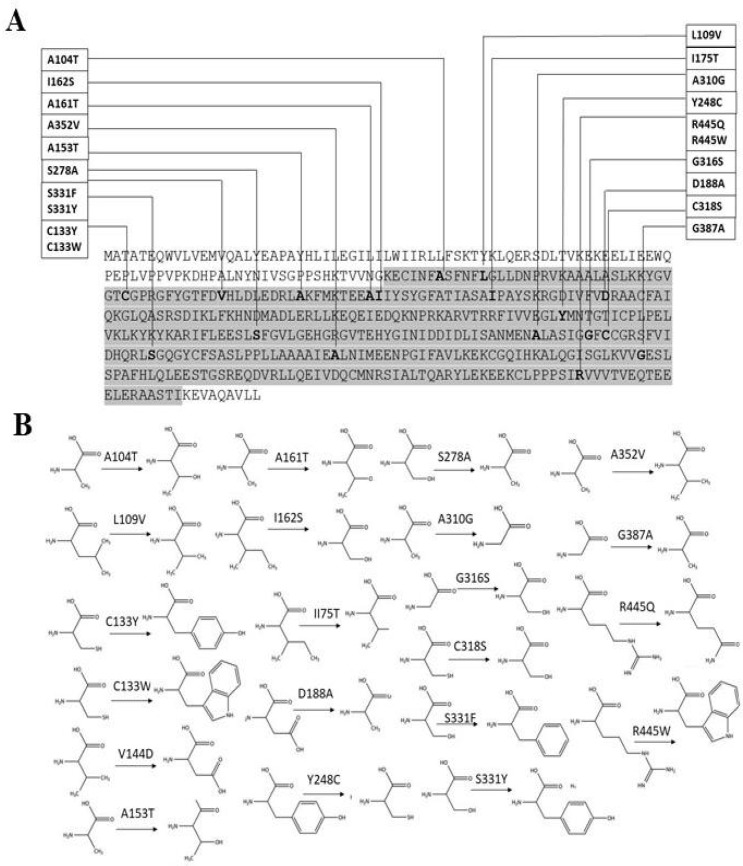
Protein mutations analysis. A: 21 identified mutations in the protein sequence showed that all the predicted mutations belong to the serine C-palmitoyltransferase activity domain of the protein; B: structural differences between selected wild type and mutant residues of SPTLC1 protein

**Indel, frameshift and UTR variants analysis**

A total of 94 UTR variants were identified. Out of which 17 variants were lying in 5’ UTR and 77 in 3’ UTR sequences (Tables 3 and 4). The UTRscan identified that 21 variant had no effect, but 55 variants were lying in the region important for open reading frame (ORF) and 1 variant c.*1302T>G in 3’UTR was found in the region associated with polyadenylation signal (PAS), cytoplasmic polyadenylation (CPE), internal ribosomal entry site (IRES) (Table 3).

**SPTLC1 structural analysis**

**3D structure modeling and evaluation**

Native human SPTLC1 model built by homology modeling based on 3a2b.1.A template showed the good overall quality and stereo-chemical properties suggesting a reliable structure (Fig. 2). The whole structure was modeled from 83-471 residues and consisted of 17 alpha and 12 beta sheets. RAMPAGE showed 376 (97.2%) residues in favored region, 9 (2.3%) in allowed region, and only 2 (0.5%) residues (Ala172 and Lys268) as outliers. However, the local model quality estimated by PROSA-web calculated the energies of residues as negative and the overall quality model of the predicted structure indicated the -9.24 z-score that lies within the characteristic range.

**Table 5:** Surface accessibility prediction scores by NetSurfP and ResQ web server for models**.**

**Position**	**Residue**	**RSA**	**ASA**	**Z-score**	**Class assigned**	**rBF**	**nBF**
104	A	0.022	2.402	0.215	B	21.03	-0.38
	T	0.031	4.244	-0.356	B	-	-
109	L	0.089	16.296	-0.912	B	22.39	-0.21
	V	0.086	13.234	-0.852	B	-	-
133	C	0.2	28.108	-2.467	B	23.41	-0.08
	Y	0.22	46.993	-2.339	B	-	-
	W	0.182	43.867	-2.631	B	-	-
144	V	0.117	17.937	0.914	B	23.54	-0.06
	D	0.105	15.188	0.997	B	-	-
153	A	0.128	14.128	0.525	B	22.14	-0.24
	T	0.156	21.609	0.577	B	-	-
161	A	0.017	1.840	0.828	B	21.90	-0.27
	T	0.018	2.441	0.737	B	-	-
162	I	0.033	6.105	0.675	B	20.65	-0.27
	S	0.035	4.102	0.607	B	-	-
175	I	0.044	8.214	0.470	B	21.16	-0.36
	T	0.047	6.477	0.275	B	-	-
188	D	0.085	12.220	-0.493	B	22.10	-0.24
	A	0.072	7.912	-0.395	B	-	-
248	Y	0.087	18.656	-0.160	B	22.43	-0.20
	C	0.092	12.917	-0.080	B	-	-
278	S	0.040	4.676	-1.360	B	21.67	-0.30
	A	0.040	4.419	-1.457	B	-	-
310	A	0.047	5.223	-2.633	B	22.10	-0.24
	G	0.045	3.534	-2.626	B	-	-
316	G	0.028	2.196	-1.564	B	20.77	-0.41
	S	0.034	3.973	-2.044	B	-	-
318	C	0.045	6.290	-0.178	B	21.33	-0.34
	S	0.037	4.301	-0.952	B	-	-
331	S	0.360	42.227	-0.624	E	24.66	0.07
	F	0.360	72.352	-0.700	E	-	-
	Y	0.376	80.394	-1.037	E	-	-
352	A	0.025	2.799	0.332	B	21.44	-0.33
	V	0.025	3.873	0.234	B	-	-
387	G	0.311	24.460	-1.840	B	29.44	0.67
	A	0.340	26.750	-1.867	E	-	-
445	R	0.041	9.389	0.259	B	20.76	-0.41
	Q	0.043	7.662	0.338	B	-	-
	W	0.047	11.328	0.068	B	-	-

RSA: relative surface area (value <0.2 (buried residues);> 0.2 (exposed residues). ASA: absolute surface area (value <25% of ASA_max_(buried); value> 25% of ASA_max_(exposed)); B: buried or E: exposed; rBF: raw beta factor; nBF: normalized beta factor.

**Protein characteristic properties analysis**

In our analysis, PredictProtein predicted that most of the residues were in buried region (Fig. 3A). Thus, we employed NetSurfP server. Most of the identified mutant residues belonged to the buried region of protein (Table 5) except Ser331. Moreover, the estimated local quality defined as the distance deviation between native and model protein residual position using support vector regression showed that most of the residues were below the cut-off value (Fig. 3B). The stability and flexibility of different parts of the model evaluated by ResQ server depicted that most of the residues belonged to the well-order structure of the protein as the calculated raw and normalized beta factor values were less than the cut-off score (Fig. 3C and Table 5). It has been observed that the mutated residues belonged to the serine C-palmitoyltransferase activity domain (Fig. 4A). Also, structural difference of amino acids revealed that substituted residues have explicit properties like size, shape, density and charges (Fig. 4B), thus would impact the stability and interaction with other molecules

**Functional analysis of mutations**

To elucidate the protein function and its association with other molecules, protein network analysis and interaction pattern has opened the avenues. Top 5 binding pockets predicted by GHECOM were graphically represented in Fig.5A.

**Protein-protein network and interaction analysis**

The STRING database exhibited 10 functional partners of SPTLC1, among which 8 were found with the confidence score >0.9 and two with score >0.99 (Fig. 5B and Table 6). Predicted interaction network has demonstrated that SPTLC2 and SPTLC3 were the strongest interaction partners with highest score (c ≥ 0.99) (Fig. 5B and Table 6) and were shown to be involved in heterodimer formation with SPTLC1 protein. We pursued our analysis to investigate the SPTLC1 protein interaction upon binding to SPTLC2. Interacting residues of SPTLC1 with SPTLC2 protein are illustrated in Fig. 6.

**Fig. 5:**
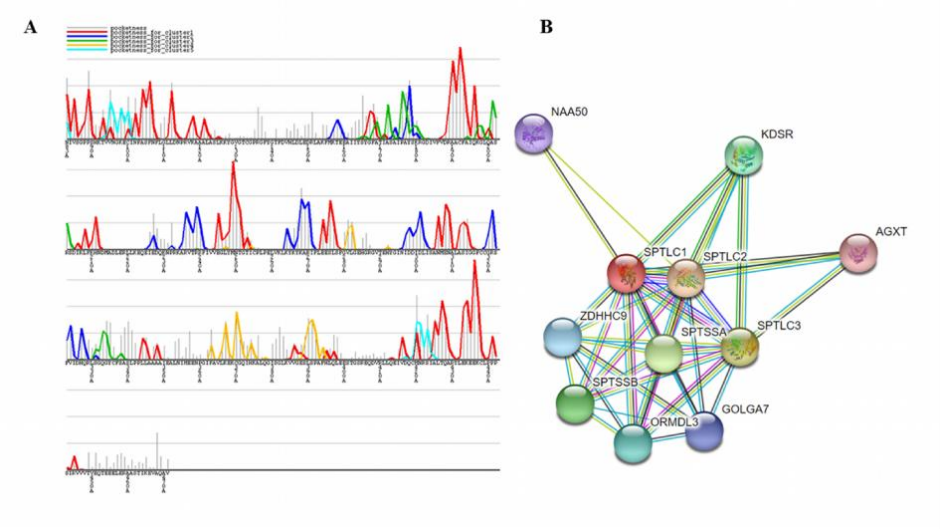
Functional analysis of mutations. A: predicted multi-scale binding pockets on protein surface representation by GHECOM; B: functional protein network analysis. STRING interaction interwork show the association of SPTLC1 with different protein partners. In the above picture circles represent the one protein and the edges represent the protein-protein interactions

**Table 6:** Predicted functional partners of SPTLC1 by STRING database.

**Node 1**	**Node 2**	**Neighbourhood** **on** **chromosome**	**Phylogenetic** **cooccurrence**	**Homology**	**Co** **expression**	**Experimentally determined interaction**	**Database annotated**	**Automated** **Text mining**	**Combined** **score**
SPTLC1	SPTLC2	0	0.526	0.74	0.27	0.925	0.9	0.931	0.996
SPTLC3	SPTLC1	0	0.526	0.733	0.27	0.921	0.9	0.928	0.995
SPTSSA	SPTLC1	0	0	0	0.049	0.329	0.9	0.864	0.99
SPTSSB	SPTLC1	0	0	0	0	0.329	0.9	0.864	0.99
KDSR	SPTLC1	0.09	0	0	0.092	0	0.9	0.652	0.967
ORMDL3	SPTLC1	0	0	0	0.128	0.462	0.9	0.282	0.961
ZDHHC9	SPTLC1	0	0	0	0.053	0	0.9	0.274	0.925
GOLGA7	SPTLC1	0	0	0	0.053	0	0.9	0	0.901
SPTLC1	NAA50	0	0	0	0.104	0	0	0.868	0.877
AGXT	SPTLC1	0	0	0	0.051	0	0.8	0.187	0.832

**Fig. 5:**
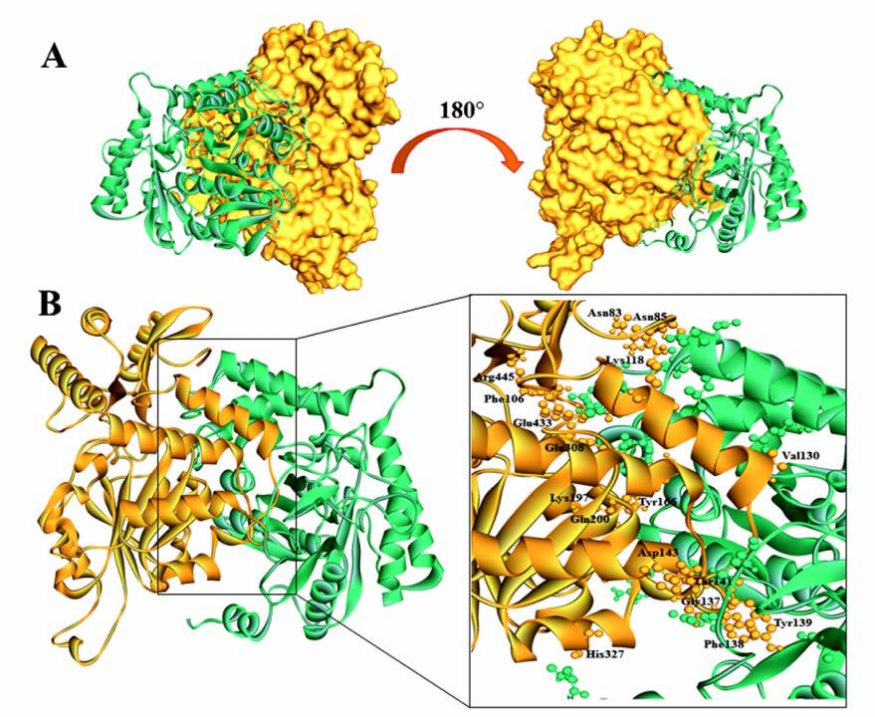
Proposed binding interaction model between wild SPTLC1 and SPTLC2 reveals the active residues of wild SPTLC1 protein. Orange represents wild SPTLC1 while green represents SPTLC2. A: illustration of interacting model and binding pocket before and after 180o rotation; B: residues of wild SPTLC1 binding pocket involved in interaction are labelled

## Discussion

Due to the continuous discovery of genetic variations, experimentally delineation of the correlation of disease associated missense variants with underlying biological mechanism is demanding. Thus, in the era of computational biology, advanced *insilico* programs exhibit reliable approach in listing out the candidate genetic variants in accordance to their deleterious impact and consequence on structure and function of corresponding proteins. The concordant analysis of prediction programs increases the prediction accuracy, and thus reduces the false positive rate.

In the present study, computational screening was done using sequence and structure homology-based programs including SIFT and PolyPhen-2. Computational pathogenic variants prediction programs review has inferred that SIFT and PolyPhen has better execution power in identifying the pathogenic variants (47), likewise supported by Gnad et al., in 2013 (48). In addition, we also incorporated PROVEAN and MutPred results for our analysis. All missense mutations were further checked for disease association. Deleterious missense mutations predicted by three of the servers were selected. The destabilizing effect in majority of the deleterious mutations gives an indication about the disturbance in the structure and function of protein. Taking in consideration the above selection scheme, the selected mutations were screened from the data sets for further analysis (Table 1).

Among all selected missense mutations (Table 1), clinical and molecular consequences of some of the mutations have been reported previously (49-53). The variant p.S331F wass found to be associated with early-onset and a severe HSAN phenotype (49, 50, 53). Additionally, p.C133W, p.C133Y and p.V144VD variations in SPTLC1 were the most examined missense mutations and observed to be the most widely recognized reason for HSAN-I (5, 51, 54, 55). Our concordant *in silico* predictions for p.C133W, p.C133Y and p.V144D mutations also revealed the high deleterious effects (Table 1).

Prediction results of SIFT Indel Classifier and PROVEAN depicted frameshift variants and indels in coding sequence of *SPTLC1* as deleterious. Likewise, UTR variants were examined to search a variant in any functional pattern endorsed by UTRsite and UTR database. The 3’UTR contains the two different polyadenylation signals that mediate the poly (A) tail synthesis (56): nuclear polyadenylation signal (PAS) and CPE element.

Native human SPTLC1 model built by homology modeling based on 3a2b.1.A template shows the good overall quality and stereo-chemical properties. Protein relative solvent accessibility gives a protein structural and functional insight (57) as due to a residual mutation the solvent accessibility can be decreased, affecting protein stability. On average, disease causing variants that are likely to destabilize the protein reside mostly at the buried region of protein (58). In our analysis, most of the identified mutant residues belonged to the buried region of protein (Table 5) except Ser331. It has also been observed that the mutated residues belonged to the serine C-palmitoyltransferase activity domain and the structural difference of amino acids revealed that substituted residues have explicit properties like size, shape, density, and charges (Fig. 4B), and thus will impact the stability and interaction with other molecules.

Predicted interaction network demonstrated that SPTLC2 and SPTLC3 were the strongest interaction partners. The SPTLC1-SPTLC2-SPTSSA complex expresses a strong preference for C16-CoA substrate, while SPTLC1-SPTLC3-SPTSSA complex uses both C14-CoA and C16-CoA substrate, with slight preference for C14-CoA (59). A study shows that *SPTLC1* mutations induce a shift in SPT substrate specificity that leads to the formation of atypical non-degradable neurotoxic sphingolipid metabolites resulting in HSAN-I (13). Study has also revealed the importance of disease-causing mutations in the active site of SPT that alters the relative positions of hydrophobic residues of both SPTLC1 and SPTLC2 subunits at dimer interface, thus affecting the enzyme activity (9, 60). Hence, it is certainly estimated that the enzymatic action of SPT would be influenced by the mutations either through the allosteric property of protein or the disturbance in the geometry of key residues present within the active site of enzyme that contributes in the recognition of substrate, or through the inadequate dimerization of the SPT monomers (61). It has been reported that in p.C133W, p.C133CY and p.V144D model, these amino acid residues do not specifically interact with the coenzyme or the substrate but lie at two closures of the loop that contact the other monomer to retain the dimer structure (61). Our study also shows that these selected residues also do not directly contact with SPTLC2 protein, but may be present around the interacting residues (Fig. 6).

Many previous comprehensive studies have shown the efficacy of consolidated computational programs for sorting detrimental variants from huge dataset (62-68). Previous studies have mentioned several physiological alterations in *SPTLC1* mutant cells, including a rise in both ER stress and potential oxidative phosphorylation (13, 14). Thus, in this study, we systematically demonstrated the computational investigation of SPTLC1 variants to study the aberrant effect of most deleterious variants affecting the structural and functional properties of protein.

In the study, we demonstrated a bioinfor-matics-based strategy for prioritizing the potentially functional SNPs from enormous set of poly-morphisms. It proposes that the combination of various computational tools may impart an alternative approach that could opt for targeting SNPs. However, the functional consequence of candidate SNPs was not experimentally evaluated. We believe that in future our provided prioritized list of potentially deleterious variants will be helpful for determine the contribution of key SNPs in disease progression.
